# Broadband cavity enhanced UV-VIS absorption spectroscopy for picolitre liquid samples†

**DOI:** 10.1039/d3an00143a

**Published:** 2023-03-15

**Authors:** Imogen M. Fermor-Worth, Catalin Chimerel

**Affiliations:** a Living Systems Institute, Faculty of Health and Life Sciences, University of Exeter Stocker Road Exeter Devon EX4 4QJ UK catalin.chimerel@unitbv.ro; b Automation Department, Faculty of Electrical Engineering and Computer Science, Transilvania University of Brasov 500036 Brasov Romania

## Abstract

Absorption spectroscopy is a widely used analytical technique due to its label-free nature, however its application to small liquid samples is hampered by the associated short absorption pathlengths, which limit sensitivity. A novel concept for the development of an ultrasensitive broadband absorption spectrometer optimised for thin liquid films is presented here. To enhance sensitivity of the absorbance measurements an optical cavity is implemented on a fibre-based absorption spectrometer (CEASpec). Light is circulated multiple times through the sample of interest to increase sensitivity. The bandwidth of the instrument is chosen by the choice of the dielectric mirrors forming the optical cavity spectra and, in this implementation, has been set to be 200 nm wide (250–450 nm). The sensing volume of the spectroscope is prescribed by the choice of optical fibres employed to deliver light to the sample, and in this implementation fibres of 400 μm in diameter were employed, giving a sensing volume of 630 picolitres for a thin film of 5 μm in thickness. Amphotericin B, a broad light absorber in the 280–450 nm region of the spectrum, was used here to prove the capabilities of the proposed cavity enhanced absorption spectroscope. Cavity enhancement factors, the equivalent pathlength increase over classical absorption spectroscopy, in the range of 200× have been achieved across a broad wavelength range.

## Abstract 2

An absorption spectrometer for small volume liquid analysis with a cavity that increases sensitivity in a broad UV-VIS range.

## Graphical abstract

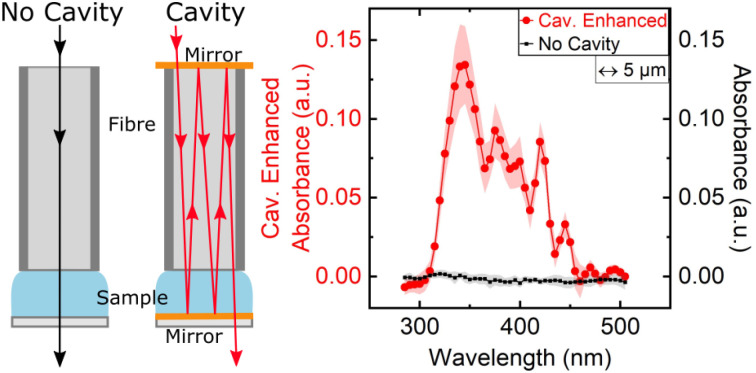

## Introduction

Absorption is a universal process which all matter undergoes when interacting with light. The individual atomic or molecular structure of a material determines which discrete wavelengths of incident light are absorbed, and therefore gives a ‘spectral fingerprint’ for every chemical. Measuring the spectral properties of the absorbance of materials has been utilised extensively across the scientific world for purposes varying from determining stellar composition^1^ to quantifying nucleic acid concentrations.^2,3^ Absorption spectroscopy is label free and universal,^4,5^ non-destructive and even removes the need for fluorescent tags,^6^ which can be time consuming, resource intensive, and modify the physical–chemical properties of specific analytes.^7,8^ Routinely used absorption spectrometers in research environments have evolved from measuring a sample solution in a centimetre sized cuvette with a corresponding volume of ∼1 ml to a ∼1 μl sample.^2^

Measuring small volumes is particularly important for applications such as nanoparticle synthesis and characterisation, drug development, and single cell omics.^4,9–12^ The sensitivity required for measuring small volumes or low concentrations with traditional absorption spectroscopy has been beyond capabilities.^13^ The limiting factor for a sample of concentration *C* is the pathlength, *l*, of light through the sample, which is directly related to the sample volume. To extend the path length through a given volume various methods have been employed, including waveguides which contain and direct light through the sample. Waveguides containing the sample, or adjacent to sensing layers, have been achieved and shown to successfully enhance the absorption capability.^14–17^ In order to further minimise the measured sample volume an optical cavity must be used so the reflected light is confined to a specific volume.

Developing cavity enhancement methods has been of interest since the 1980's with the development of Cavity Ring Down Spectroscopy (CRDS), which measures the decay of light in an optical cavity interacting with a sample.^18^ This technique has been extensively used in gas detection and for some liquids phase samples.^19–23^ Combining CRDS with evanescent wave spectroscopy has enabled studies of thin films of solution, and proven effective at measuring surface interactions such as adsorption.^24–27^ A drawback of CRDS is that it is typically limited to long pathlengths (centimetres to metres) by the sensitivity and expense of detection instrumentation, especially for shorter intra-cavity distances.^4^ The development of CRDS sprouted a number of remarkable related techniques such as phase shift cavity ring down spectroscopy in the 90's,^28–30^ whispering gallery mode-based sensors,^31,32^ and Cavity Enhancement Absorption Spectroscopy (CEAS). CEAS measures the time integrated change in light intensity transmitted through the sample,^24,28,33^ this has the benefit of reduced instrument sensitivity requirements so it can be more readily adapted to shorter cavity lengths. Using CEAS to measure the absorbance of a solution allows the detection of small volume samples whilst maintaining low concentrations. Cavity enhancement absorption spectroscopy for small liquid volumes was approached by miniaturising the optical path through a glass cuvette (20 cm to 2 mm) encompassed in an optical cavity,^34,35^ however with miniaturisation the enhancement factor (429×) also reduced (100×), due to enhanced losses associated with the miniaturised cuvette.^35^ However this miniaturisation has allowed subsequent integration of CEAS and analytical techniques such as HPLC,^36^ stopped-flow kinetics^37^ with sensing volumes on the millilitre to microliter scale, and evanescent wave based techniques.^30,38^

Furthermore CEAS has also been employed in microfluidic devices on volumes of approximately 90 nl, where an enhancement factor of 102× has been achieved.^39^ Due to scattering at the material interfaces further reducing the microfluidic sensing volume to 10 nl reduces the enhancement factor to 28×.^40^ To avoid scattering, micro-cavities directly in contact with the sample were developed. Optical fibres have been employed to act as a waveguide between the cavity mirrors and the sample to generate a CEAS with an enhancement factor of 9× in volumes ∼200 pl.^9,10^ Using a plano-convex cavity configuration with volumes down to 50 fl an increased cavity enhancement factor of 266× was achieved.^4^ However, the plano-convex geometry is tuned for a single resonant wavelength at ∼640 nm and measurements over a broad spectral range are difficult to achieve as they require the adjustment of the cavity geometry *via* a piezo actuator. Moreover, the fabrication of the microcavities involves focussed ion beam micromilling of glass surfaces which is a technically challenging and costly procedure.^11,41^ Here we are proposing a novel optical fibre-based approach for CEAS which allows for broadband measurements and can achieve an enhancement of 200× on volumes as low as 630 pl, with the ability to further minimise the sensing volume.

## Experimental section

### Design of CEASpec instrument

**Fig. 1:**
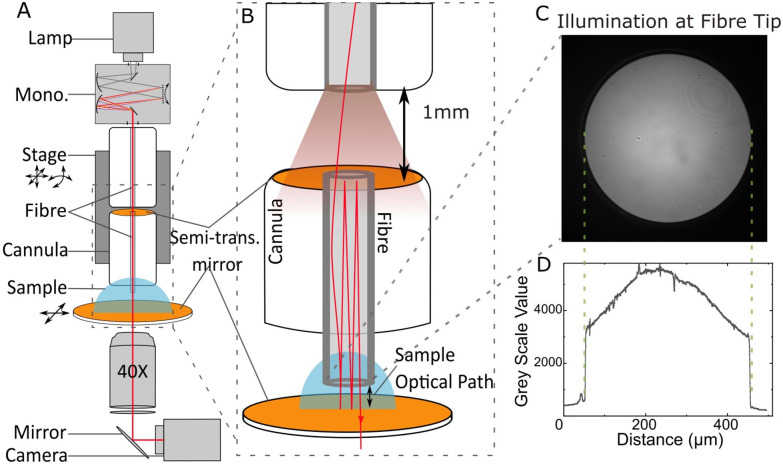
Schematic of CEASpec broadband cavity enhanced UV-VIS absorption spectroscope. (A) Arrangement of the principal components of the broadband cavity enhanced absorption spectroscope. From top: laser driven light source (lamp); optical fibre coupled monochromator (mono.); multidirectional stage; optical fibre cannula with S1 Optics GmbH dielectric thin film coating (top semi-trans. mirror); sample solution; UV cover slip with LAYERTEC GmbH dielectric thin film coating (bottom semi-trans. mirror); quartz 40× objective lens; UV-enhanced mirror; sCMOS camera. Inset (B) detailed arrangement of optical fibre and mirror positions, showing the light path trapped inside and through the optical cavity. A 1 mm gap between the fibre tip delivering light from the monochromator and the canulae tip coated with the semi-transparent mirror is highlighted. (C) Image of the illumination at the fibre tip. (D) Light Intensity profile across the equatorial line of the illuminated fibre tip displayed in panel (C).

The design of the broadband cavity enhanced UV-VIS absorption spectroscope (CEASpec) is presented in Fig. 1. In brief, a liquid thin film was entrapped between two planar mirrors, circulating the light multiple times through the sample, enhancing the absorption measurement. Light was delivered to the liquid thin film, supported on a quartz cover slip coated with a semi-transparent dielectric mirror, with an optical fibre held (FG400AEA Thorlabs Inc.) in a 12 mm long cannula. The optical fibre cannula tip was submerged into the solution entrapping the thin liquid film between the fibre tip and the microscope quartz coverslip (CFS-2520, UQG Optics Ltd). Cannulas were made with optical fibre lengths fixed into ceramic ferrules using an epoxy glue (EPO-TEK 353ND, Epoxy Technology Inc.) leaving approximately 2 mm of exposed bare fibre tip through one end. The other end of the ferrule was polished using diamond lapping sheets and coated with a semi-transparent dielectric mirror. The two mirrors (Fig. 1B) generated a planar Fabry–Pérot cavity that circulates light multiple times through the thin film in order to enhance light absorbance. The mirror on the cannula was a high reflectivity broadband (250 nm to 450 nm) dielectric thin film (CM-B) developed by S1 Optics GmbH (SFig. 4†). The mirror on the top surface of the coverslip is a custom design LAYERTEC GmbH dielectric mirror with a reflectivity greater than 99.5% over a spectral interval ranging from 250 nm to 550 nm. The fibre cannula position was manipulated and perpendicularly aligned to the surface of the coverslip with 1 μm accuracy, using a combination of manual and piezoelectric stages that allow xyz control and pitch and tilt control, with 10 arcsecond resolution. A UV-IR Laser Driven Light Source (EQ-99X LDLS, Energetiq) with power according to the manufacturer of approximately 10–20 mW mm^−2^ nm^−1^ sr^−1^ across the relevant wavelengths, coupled to a monochromator (Cornerstone Oriel 130, Newport Corporation) provides monochromatic light to the cavity. The fibre coupled monochromator was employed to selectively transmit light into the cavity and sample solution one wavelength at a time and minimize the amount of radiation delivered to the sample. A multimode UV-Vis optical fibre of 400 μm core diameter (FG400AEA Thorlabs Inc.) delivered the selected monochromatic light with a power of approximately 16 μW, measured at 350 nm (PM100D S150C Thorlabs Inc.) into the cavity. In order to avoid damaging the mirror coating on the back of the cannula a 1 mm spacer was introduced when coupling the optical fibre, delivering light in the cavity, to the cannula supporting the mirror (Fig. 1B, top panel). Light was collected from the cavity with a quartz objective lens (Ultrafluar 40×/0.6 Glyc, Carl Zeiss Ltd). The light was then directed by a UV-Enhanced Aluminium Mirror (PF10-03-F01, Thorlabs Inc.) into a sCMOS camera (Kuro 2048B, Teledyne Princeton Instruments), used to obtain a two-dimensional image of the transmission of light through the optical cavity at each wavelength, allowing a spectrum to be created. The monochromator was controlled by LabVIEW code, the camera by LightField, the piezo stage by Thorlabs Open-Loop Piezo Controllers (V2.4.0). Positioning and alignment were aided by a set of two video microscopes (Dino-Lite AM4113ZT(R4)) imaging the fibre tip approaching the sample from two orthogonal directions.

### Experimental procedure of CEASpec

Measuring the absorbance spectrum of a solution with the CEASpec starts with alignment of the fibre cannula tip. A 400 μm fibre cannula with a dielectric film mirror coating was inserted into the CEASpec. The fibre was aligned to be perpendicular to the second mirror (Fig. 1) and optimised iteratively for maximum cavity enhancement. Subsequently, the thickness of the thin film of solution is fixed. Using Milli-Q water deposited onto the second mirror as a temporary solution, the tip of the fibre cannula was brought into contact with the second mirror. Using the piezo control the fibre tip was lifted on the *z* axis to the desired height of 20 or 5 μm away from the second mirror substrate and the objective lens was focussed on the fibre tip in this position. This was followed by a sequence of solution changes and scan measurements. In order to change solutions, the fibre tip was raised to a 20 μm height above the mirror and using lint free tissues and compressed air the surface was dried. First a blank solution of 20 mM Tris–HCl (T60040, Melford – H/1200/PB17, Fisher Scientific) 0.25 M NaCl (S/3160/60, Fisher Scientific) (pH 7.4) was deposited, and the fibre was then repositioned at the desired distance and transmission spectrum acquired. Subsequently, a droplet of the sample solution of Amphotericin B solution (A2942, Sigma-Aldrich) diluted in the blank solution to the desired concentration of 25 μg ml^−1^ was deposited and scanned using the same technique, and this process repeated for the desired number of samples at increasing concentrations (50, 75, 100, 125 μg ml^−1^). The scan constituted of the monochromator increasing stepwise wavelength output from 280 to 500 nm with a step of 5 nm, pausing at each for a measurement. At each wavelength, a trigger (DAQ BNC 2110, National Instruments) prompted the camera software to capture a frame with an exposure time of 20 ms. For each droplet, the scan was repeated three times and averaged. Each scan takes approximately 60 seconds to perform.

When comparing classic *versus* enhanced absorption, the cavity was eliminated by replacing the optical fibre cannula with one lacking the dielectric mirror coating. The repeats consisted of three uncoated cannulas and three coated cannulas used, and for each cannula the transmission measurements for the blank and sample solution were repeated three times and averaged to find the absorbance spectrum.

### Data analysis

The absorbance spectra, *A*, was quantified using the ratio of light transmitted through the blank solution, *I*_0_ and the sample solution, *I* as in eqn (1).1
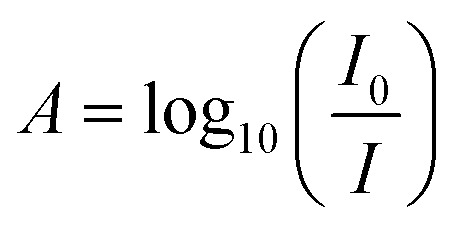


To determine the intensity of light through the solutions, cross sections of the image stacks were processed using LightField software, averaging pixel intensity over a defined illuminated area. All images were background subtracted with an average pixel value of 100.6 measured in the absence of light. The intensity at each wavelength per sample was found by averaging the intensity for the corresponding frame across three repeated scans. The average intensity per wavelength for blanks and drug samples were then inputted in eqn (1). Standard deviation for the averages between repeat scans were determined.

Cavity enhancement factor (CEF) is widely used to determine the quality of the cavity^4^ and is a gauge of a cavities performance *versus* an equivalent single-pass measurement. Here the CEF of the cavity was determined using eqn (2).2
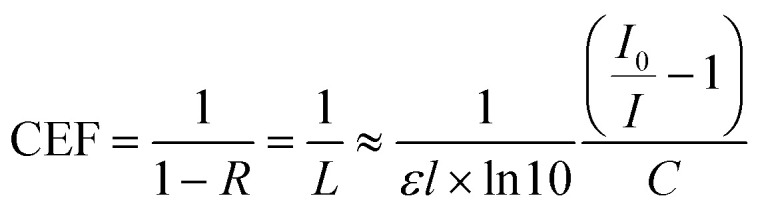
where *L* is the intrinsic loss of light in the cavity, *ε* is the molar attenuation coefficient of the sample, *l* is the pathlength, *C* is the molar concentration, 
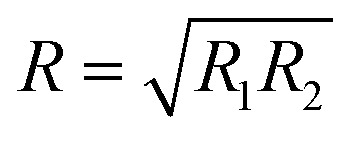
 where *R*_1_ and *R*_2_ are the reflectances for non-identical mirrors, and the other terms are as before. This holds assuming a system with losses only from the semi-transparent nature of the mirrors that form the cavity, and the light is plane-wave in nature.^42^

3 *A* = *εlC*

The molar attenuation coefficient of Amphotericin B sample solution was determined using the Beer–Lambert law (eqn (3)). For a range of wavelengths (180–500 nm) the measured the absorbance spectra of five concentrations of the solution (25, 50, 75, 100, 125 μg ml^−1^) was determined using a NanoDrop 2000c Spectrophotometer (Thermo Fisher Scientific). The absorbance *versus* concentration for each wavelength was fitted with a linear regression, and the gradient value was used in a rearranged Beer–Lambert law to find attenuation coefficient.

The concentration at the limit of detection, *C*_LOD_, minimal detectable absorption loss, *α*_min_, and minimal detectable change of absorption cross section, *σ*_min_, are also significant metrics for the capabilities of cavity enhanced spectrometers. Here the *C*_LOD_ was calculated using the methodology discussed by Loock and Wentzell,^43^ and *α*_min_ and *σ*_min_ are defined as in Loock *et al.*:^22^

4 *α*_min_ = *ε* × ln 10 × *C*_LOD_

5 *σ*_min_ = *V*_det_ × *α*_min_

where *V*_det_ is the volume of the sensing volume.

## Results and discussion

Delivering light to samples with optical fibres has facilitated modern absorption spectroscopy by reducing sample volume from the traditional 1 cm cuvette. Fibre based spectroscopes (*e.g.* NanoDrop, DeNovix, Implen) are now ubiquitous in broad research settings with biomedical and molecular biology application. Here we are demonstrating a potential methodology to enhance these devices by introducing a multimode optical cavity to further increase their sensitivity. Building multimode optical cavities that accommodate a broad range of wavelengths for analysis of small liquid volumes has been attempted before with limited success in terms of amplification capabilities^9,10^ due to their current cavity form. Here we are applying planar mirrors directly onto fibre ends, significantly reducing light losses in the cavity and enhancing sensitivity.

### Classic *vs.* cavity enhanced absorption

**Fig. 2:**
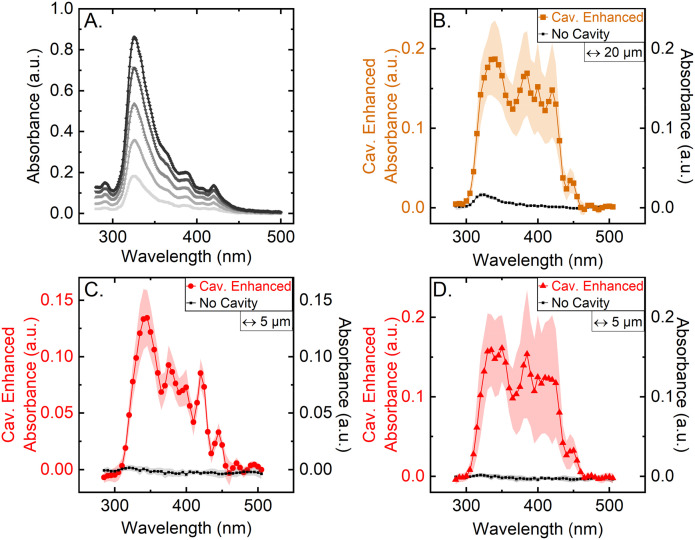
Classic *versus* cavity enhanced absorption. (A) NanoDrop 2000c spectrophotometer absorbance measurements of Amphotericin B solution at five concentrations (25–125 μg ml^−1^) with a pathlength of 1000 μm. (B) Absorbance spectra of a 20 μm thin film of Amphotericin B solution of 125 μg ml^−1^, characterised with a 400 μm multimode core fibre, as a single pass measurement (no cavity), and with the optical cavity in place (cavity). The cavity spectra represents the average of 4 independently built optical cavities, with the highlighted area representing the standard deviation between measurements. (C) Absorbance spectra of a 5 μm thin film of Amphotericin B solution of 125 μg ml^−1^, characterised with a 400 μm multimode core fibre, as a single-pass measurement (no cavity), and with the optical cavity in place (cavity). The highlighted area represents the standard deviation in the measurement. In the case of the cavity this is over 8 independent experiments measured over 2 weeks without any adjustment to the alignment of the optical cavity. (D) Average over 4 independently built optical cavities for the absorbance spectra of a 5 μm thin film of Amphotericin B solution of 125 μg ml^−1^, characterised with a 400 μm multimode core fibre, as a single-pass measurement (no cavity), and with the optical cavity in place (cavity). (a.u.) stands for arbitrary units.

To demonstrate the power of cavity enhancement, the single-pass absorbance is directly compared to the cavity enhanced absorbance measured using the CEASpec. For this purpose, a commercially available single-pass benchtop spectrometer was used to measure the absorbance of the antimicrobial drug Amphotericin B in solution at a range of concentrations (Fig. 2A). The typical pathlength of single-pass benchtop spectrometers is in the range of 1 mm, giving an approximate sensing volume of 0.3 μl. In comparison, the equivalent single-pass pathlength in the CEASpec is as low as 5 μm giving a sensing volume of 630 pl. At these low sensing volumes, and pathlengths, the use of single-pass absorbance spectroscopy is not feasible. To highlight this limitation Amphotericin B solution was measured with the CEASpec at a concentration of 125 μg ml^−1^, with and without the cavity (Fig. 2B–D). In Fig. 2B the, 20 μm thick, thin film of Amphotericin B aqueous solution had a sensing volume of 2500 pl and a significantly reduced classic absorbance spectrum. Here it is important to note that the measure of the error is given by the implementation (alignment) of the optical cavity. The trace displayed in Fig. 2B is an average over 4 repeats each employing a distinct cavity implementation, hence slightly different optical cavities lead to different cavity enhancement factors and therefore the relatively large standard deviation.

Furthermore the thickness of the thin film was further reduced to 5 μm (Fig. 2C and D) and resulted in a sensing volume of 630 pl. Here the 325 nm absorbance peak, measured with classical absorbance, is in the noise of the baseline bringing single path absorbance to the limit of its sensitivity. On the other hand, when employing cavity enhancement the absorbance spectra is enhanced by a factor of ∼50× for 5 μm thin films at 325 nm, demonstrating how cavity enhancement can aid absorbance spectroscopy in low sample volumes. It also important to note that here the variability in Fig. 2D is generated by 4 distinct implementations of the optical cavity. If one optical cavity is chosen (Fig. 2C) the standard deviation of the measurements is significantly smaller and also stable over time. The measurements displayed in Fig. 2C were measured over a 2 weeks interval from 8 distinct preparations. Hence, in a real-world scenario, once aligned a specific CEASpec would be comparable in errors with the correspondent classic absorption spectrometer. The cavity enhancement factors could be determined and applied as means of calibration for each cavity setup.

### Quantifying enhancement with CEASpec

**Fig. 3:**
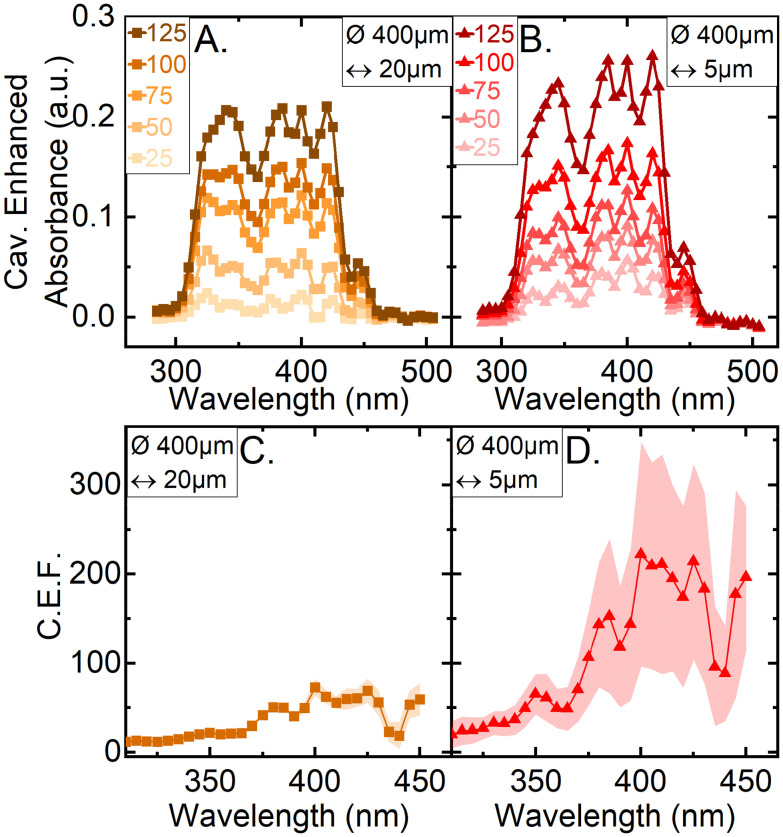
Spectral cavity enhancement. Cavity enhanced absorbance spectra of Amphotericin B solution at five concentrations (25–125 μg ml^−1^) with optical cavity in place. Thin films of Amphotericin B solution of 20 μm (A) and 5 μm (B) characterised with 400 μm diameter optical fibre. (C & D) The cavity enhancement factor for a 400 μm diameter fibre for thin films of 20 μm (C) and 5 μm (D). The values represent the average of four independent optical cavity experiments, with the shaded area representing the standard deviation in each measured point. (a.u.) stands for arbitrary units.

To evaluate the improvements to absorption spectroscopy the cavity enhancement must be quantified. One such measure is the cavity enhancement factor (CEF), which is commonly used as a metric of cavity-enhanced instruments capabilities and has been detailed in the Experimental section. By measuring the absorbance of Amphotericin B solution at a range of concentrations, the CEF was found. The absorbance of a range of concentrations (25–125 μg ml^−1^) of the Amphotericin B solution were measured for two solution thin film thicknesses: 20 μm and 5 μm. A representative example set of absorbance spectra parametrized by the Amphotericin B concentration are shown in Fig. 3A & B. Plotting the values of 
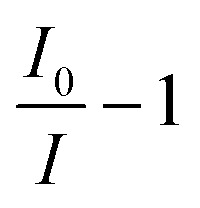
*vs.* molar concentration in the linear regime (<100 μg ml^−1^), for each measured wavelength, allows the determination of CEF by using a linear fit through the origin as described by eqn (2). The average CEF values for both thin films of 20 and 5 μm, are displayed in Fig. 3C & D. The standard deviation is calculated from four independently built optical cavities. Between 380 and 450 nm the cavity enhancement factors are equal within error, which is in line with the theoretical CEF values for mirrors of flat reflectance spectrum, as we have in our system. The lower enhancement factors below 380 nm can be attributed to the second order diffraction peaks interfering with our measurements (SFig. 2†). To analyse the amount of light present in the second order diffraction peaks we used a 550 nm longpass filter (FEL0550, Thorlabs) installed in front of the camera. The amount of light in the second order peaks ranges from a maximum 71% of the total transmitted light through the cavity at 285 nm and decreases as the wavelength is increased (SFig. 3†). At 380 nm for example only 5.6% of the transmitted light is contained in the second order peak of the spectrum. The second order peak significantly offsets the light intensity transmitted through the cavity and hence artificially reduce the absorbance and the CEF below 380 nm (Fig. 2). Notably the second order peaks do not affect the shape of the single pass spectrum measured with CEASpec (Fig. 2A & B) in the absence of the optical cavity. When present the cavity acts as a long pass filter which further reduces the relative amount of light in the first order *vs.* the second order diffraction peaks. Further corrections could involve background subtraction methods to eliminate the effect of the second order peaks or the use of monochromatic light sources such as light emitting diodes.

### Walk off losses at the fibre edges, mirror reflectivity and cavity enhancement

In contrast with the single-pass absorbance spectrometer in the CEASpec the absorbance did not linearly scale with equivalent single-pass pathlength. The magnitude of cavity enhanced absorbance on a 5 μm thin film (Fig. 2D) remained equal within error with the magnitude of cavity enhanced absorbance on a 20 μm thin film (Fig. 2B). The cavity losses are generated by the divergent light exiting the optical fibre (SFig. 1†) and consist of light that is reflected by the planar mirror and not returning into the optical fibre. For optical fibres of 0.22 numerical aperture, assuming a uniform beam profile, at 5 μm equivalent pathlength, theoretical cavity losses stand at 1.7% per cavity cycle, and for 20 μm equivalent pathlength at 6.4% per cavity cycle (ESI Section S1†). Reducing the amount of light loss when measuring thinner films improves the cavity and therefore the enhancement capabilities as illustrated when comparing 5 μm *vs.* 20 μm thin films. Furthermore, light transmitted through optical fibres has a higher intensity at the fibre core centre, which further reduces these losses. For example, using the light profiles imaged at the fibre tips (Fig. 1C and D) we estimated the light lost from the cavity at 5 μm equivalent pathlength is reduced from 1.7% to 0.6% per cavity cycle. These losses amount to 99.4% transmission efficiency of the light passing through the optical cavity, nonetheless these values are an estimation and not an actual measurement. These losses are coupled with the reflectance efficiency of the two mirrors used to build the optical cavity. The mirror coated on the quartz glass slide has a measured reflectance spectrum >99.5% ranging from 300 and 500 nm but reaches 99.75% at wavelengths greater than 350 nm (SFig. 5†). The second mirror, which is deposited on the ceramic ferrule and the back of the optical fibre, has an unknown reflectance curve. Indicative of its reflectance spectrum are the values measured for this particular coating, deposited on a quartz slide, which are on average >95% but reach 98% at several points between 300 and 500 nm (SFig. 4†). This reflectivity curve is measured in air at 8° angle of incidence, and not in the fibre core, so only indicative of the actual reflectance within the cavity. Using these indicative values, the expected Cavity Enhanced Factor (CEF) should range between 20× and 100×. For a 5 μm film the average CEF between 380 and 450 nm reaches 200×. This exceeds our theoretical prediction of 100×, indicating a possible underestimation of reflectivity on the optical fibre mirror coating.

### Limit of detection

The calculated concentration at the limit of detection, *C*_LOD_, across four different optical cavities, on a 5 μm thin film of Amphotericin B solution (Fig. 3D) is 20.3 μg ml^−1^ (22 μM) (SFig. 6†). This has been determined for the absorbance peak at 340 nm, a defined peak for Amphotericin B absorbance, as shown in Fig. 2A. This *C*_LOD_ means that the absorbance of a 20.3 μg ml^−1^ solution of Amphotericin B is reliably detectable with a sensing volume of 630 pl. The minimal detectable absorption loss, *α*_min_, was determined to be 2.3 cm^−1^ and the minimal detectable change of absorption cross section, *σ*_min_, was 145 μm^2^.

## Conclusions

A broadband UV-Vis absorption spectrometer using cavity enhancement has been developed for liquid sample analysis with sensing volumes down to 630 pl on a 5 μm thin film. Cavity enhancement factors up to 200× have been achieved, along with a significant reduction in the concentration limit of detection over traditional single pass measurements. Comparing figures of merit for the CEASpec with those determined for other cavity enhancement analytical methods^22,37,44^ it is evident that the minimal detectable absorption loss in this work is higher than those previously achieved elsewhere. Nevertheless the emphasis of this work is the broadband nature of the instrument, leading to a more widely applicable analytical tool. In creating a broadband UV/Vis instrument the reflectance of the mirrors had to be reduced, and correspondingly the limit of detection is not as low as those possible for single wavelength tools. In spite of this, the low sampling volume in the CEASpec results in a minimal detectable change of absorption cross section of comparable magnitude to previous works.^22^ The low sampling volume and broadband nature of the CEASpec has resulted in an analytical tool which could be suited to a wide range of applications.

Enhancement factors are determined by cavity losses. Contributors include the divergence of the light, non-parallelism of the cavity mirrors, or scattering in the sample or sample holder. Non-parallelism of the cavity mirrors remains our most difficult hurdle, the challenge of alignment of the fibre tip orthogonally to the mirror coated slip results in the deviation in the enhanced absorbance measured. The robustness of the instrument can be further improved by the use of motorised stages for full automated fibre alignment. Nevertheless, as shown in Fig. 2C when one cavity alignment is set the measurement errors are drastically reduced providing the CEASpec as good candidate to upgrade current fibre-based spectroscopes.

The bandwidth, or amplification window, of the instrument was determined by the light source and choice of dielectric mirrors forming the optical cavity, which has been designed to be 200 nm wide (250–450 nm) in order to suit a variety of solute “molecular fingerprints”. Amphotericin B was used as a model system due to its absorbance spectrum that spans the CEASpec amplification window. The amplification window can be further modified to suit other analytes by adjusting the reflectance window of the dielectric mirrors, and selecting an appropriate light source. We have chosen to use the LDLS UV-IR source in this case due to the broad and relatively flat output, this could be exchanged for LEDs, deuterium or halogen lamps depending on the desired bandwidth and wavelengths of interest.

In conclusion the principal components of the CEASpec could be readily integrated into currently available spectroscopes, which would be a cost effective way to significantly increase the sensitivity of such instruments.

## Author contributions

Imogen M. Fermor-Worth: investigation, methodology, software, formal analysis, writing – original draft; Catalin Chimerel: conceptualization, methodology, formal analysis, funding acquisition, resources, supervision, writing – original draft.

## Conflicts of interest

There are no conflicts of interest to declare.

## Supplementary Material

AN-148-D3AN00143A-s001
