# Supplementary material for: Broadband cavity enhanced UV-VIS absorption spectroscopy for picolitre liquid samples
Source: Analyst. 2023 Mar 15;148(8):1797–804. doi: 10.1039/d3an00143a (PMC10089060; doi:10.1039/d3an00143a)
Supplement: AN-148-D3AN00143A-s001 [file AN-148-D3AN00143A-s001.pdf]

Electronic Supplementary Material (ESI) for Analyst.
This journal is © The Royal Society of Chemistry 2023

Supporting Information

Broadband Cavity Enhanced UV-VIS Absorption Spectroscopy for Picolitre Liquid Samples

Imogen M. Fermor-Worth[a], Catalin Chimerel[ab]

[a] Living Systems Institute, Faculty of Health and Life Sciences, Stocker Road, University of Exeter, Exeter, Devon, UK EX4 4QJ

[b] Automation Department, Faculty of Electrical Engineering and Computer Science, Transilvania University of Brasov, 500036 Brasov, Romania

Contents

Section S1. Calculating the amount of light that escapes the re-entry into the optical fibre upon mirror reflection. .. S2

Section S2. Supplementary Figures. S3

Supplementary Figure 1. Ray diagram of light exiting optical fibre........ S3

Supplementary Figure 2. Spectral characterisation of the light input in CEASpec S3

Supplementary Figure 3. Transmission curve through the optical cavity of CEASpec S4

Supplementary Figure 4. Reflectance values for the dielectric coating deposited on the fibre end S4

Supplementary Figure 5. Reflectance values for the dielectric coating deposited on the UV cover slip. S5

Supplementary Figure 6. Determining the concentration at the limit of detection S5

Section S1. Calculating the amount of light that escapes the re-entry into the optical fibre upon mirror reflection.

Light exiting an optical fibre does so at a maximum angle, θ, related as in in Equation (1) to its numerical aperture (NA), and n_0 the refractive index of sample solution.

$$\sin\theta = \frac{NA}{n_0} \tag{1}$$

When reflecting from the dielectric mirror cover slip, some light will not re-enter the fibre, but escape from the cavity. The maximum angle at which light can exit the fibre and be returned to the fibre, and thus stay within the cavity is σ in Supplementary Figure 1. The radius, r, of the fibre can be separated into two concentric rings, the inner ring of radius λ where all of the incident (light will be reflected back into the fibre, and thus remain in the cavity. The outer ring of width x is the light exiting the fibre between the angles σ and θ, and will not be reflected back into the cavity. Assuming a flat intensity profile, the ratio of the areas of the outer ring and the total fibre tip gives the loss of light intensity upon every cycle through the cavity, shown in Equation ().

$$\left(\frac{\lambda}{\lambda + x}\right)^2 = \left(\frac{1}{\frac{2l\tan\theta}{r} + 1}\right)^2 \tag{2}$$

For the 400 µm core diameter optical fibres used in the CEASpec, the calculated light lost with a solution thin film, l, of 5 µm is 1.7% per cycle, and with a film of 20 µm the loss is 6.4% per cavity cycle. Using the real intensity profile (Figure 1C), imaged at the fibre tip, and superimposed over the predicted light escape rings a calculated escape rate of 0.6% is measured for a 5 µm thin film.

Section S2. Supplementary Figures.

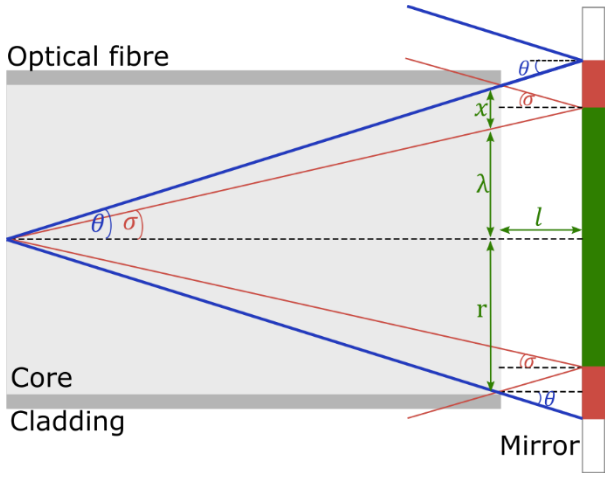

Supplementary Figure 1. Ray diagram of light exiting optical fibre. Optical fibre of radius r with a liquid thin film thickness, l, measured between the fibre tip and the mirror. The ray at angle θ is the maximum angle at which light will exit the fibre. The angle σ is the maximum angle at which light can exit the fibre and be reflected and re-enter the fibre. The ratio of the areas corresponding to rays exiting the fibre at these angles correspond to the light losses per cavity cycle.

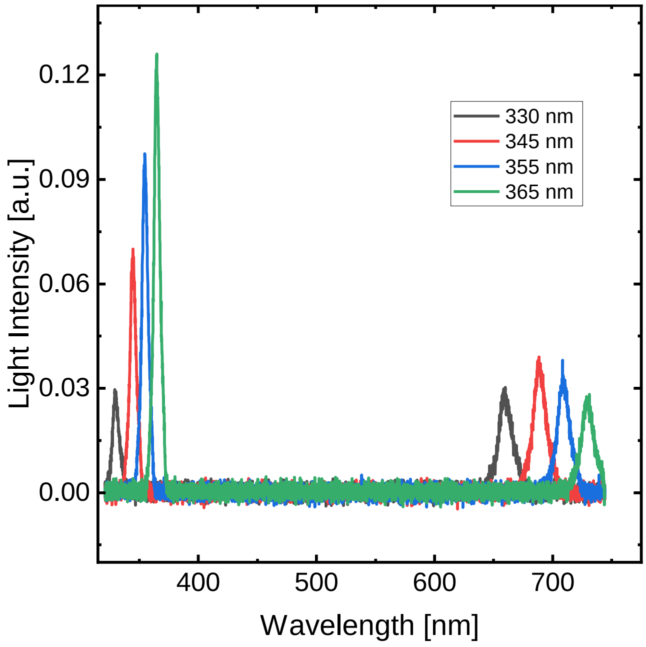

Supplementary Figure 2. Spectral characterisation of the light input in CEASpec characterised with a Thorlabs CCS100/M spectrometer.

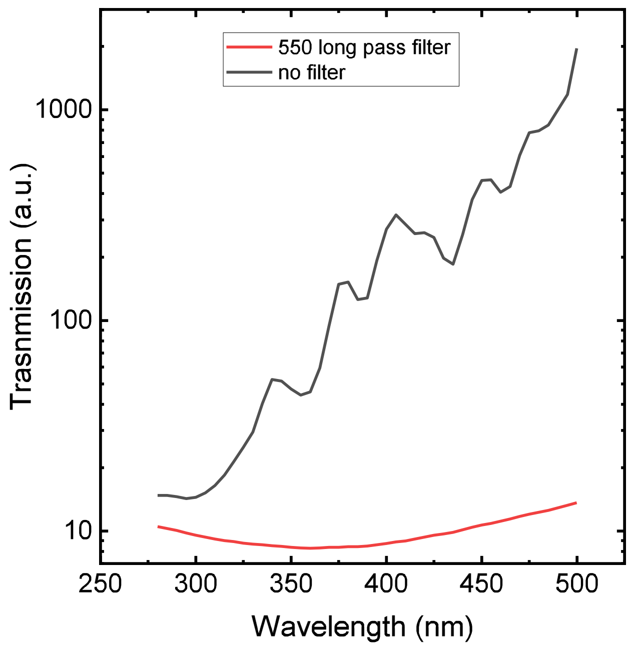

Supplementary Figure 3. Transmission curve through the optical cavity of CEASpec as detected by the imaging camera in the presence (red) and absence (black) of a 550 nm long pass filter.

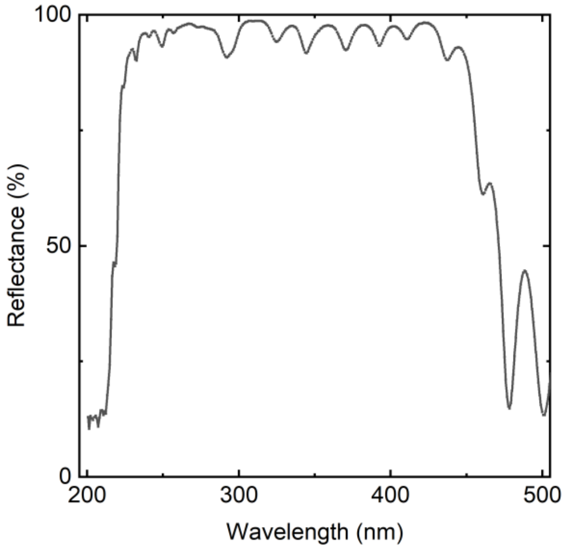

Supplementary Figure 4. Reflectance values for the dielectric coating deposited on the fibre end, Data provided by S1 Optics, measurement at 8° angle of incidence of coating on flat optical substrate.

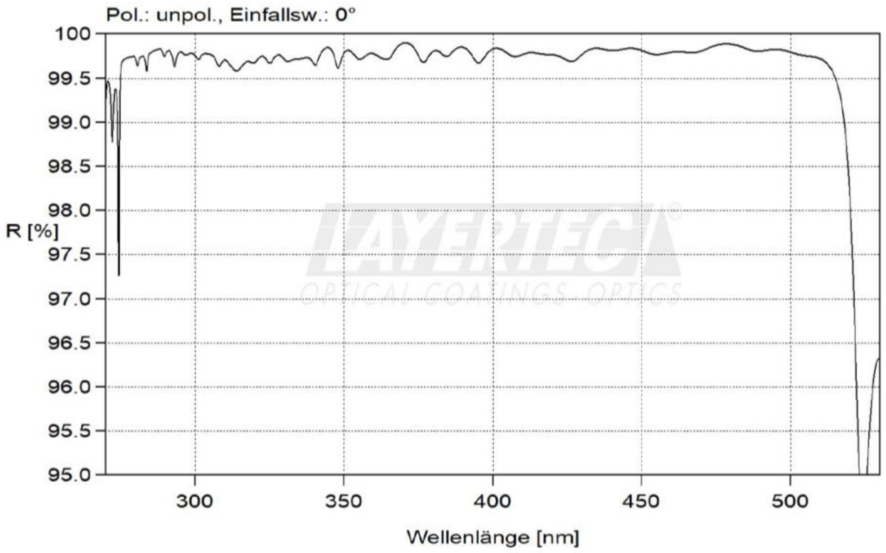

Supplementary Figure 5. Reflectance values for the dielectric coating deposited on the UV cover slip. Data provided by LAYERTEC GmbH, determined as the inverse of the transmission at 0° angle of incidence.

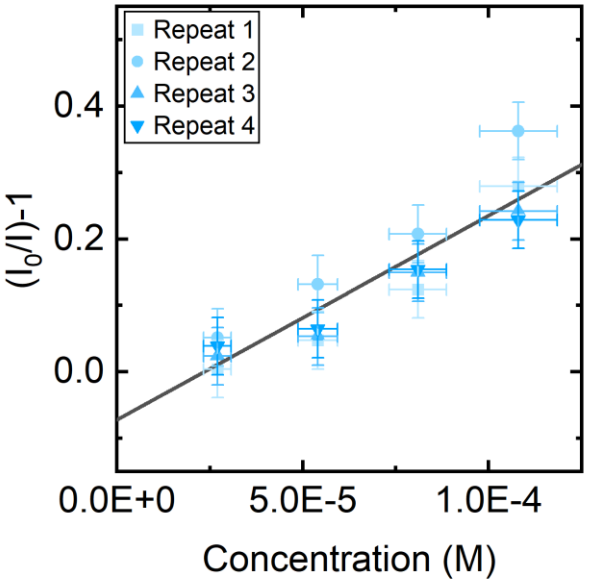

Supplementary Figure 6. Determining the concentration at the limit of detection from 4 different optical cavities. Resultant limit of detection 22 µM.
